# Remission from antipsychotic treatment in first episode psychosis related to longitudinal changes in brain glutamate

**DOI:** 10.1038/s41537-019-0080-1

**Published:** 2019-08-01

**Authors:** Kate Merritt, Rocio Perez-Iglesias, Kyra-Verena Sendt, Rhianna Goozee, Sameer Jauhar, Fiona Pepper, Gareth J Barker, Birte Glenthøj, Celso Arango, Shôn Lewis, René Kahn, James Stone, Oliver Howes, Paola Dazzan, Philip McGuire, Alice Egerton

**Affiliations:** 1 Department of Psychosis Studies, Institute of Psychiatry, Psychology & Neuroscience, De Crespigny Park, London, SE5 8AF UK; 2 Department of Neuroimaging, Centre for Neuroimaging Sciences, Institute of Psychiatry, Psychology & Neuroscience, De Crespigny Park, London, SE5 8AF UK; 3 Center for Clinical Intervention and Neuropsychiatric Schizophrenia Research, CINS, & Center for Neuropsychiatric Schizophrenia Research, CNSR, Mental Health Center Glostrup, University of Copenhagen, København, Denmark; 4 CIBERSAM: Centro Investigación Biomédica en Red Salud Mental, Santander, Spain; 5 Institute of Brain, Behaviour and Mental Health, Manchester Academic Health Sciences Centre and Manchester Mental Health and Social Care Trust, Manchester, M13 9PL UK; 6 Department of Psychiatry, Icahn School of Medicine, New York, USA

**Keywords:** Biomarkers, Schizophrenia, Psychosis, Psychosis, Schizophrenia

## Abstract

Neuroimaging studies in schizophrenia have linked elevated glutamate metabolite levels to non-remission following antipsychotic treatment, and also indicate that antipsychotics can reduce glutamate metabolite levels. However, the relationship between symptomatic reduction and change in glutamate during initial antipsychotic treatment is unclear. Here we report proton magnetic resonance spectroscopy (1H-MRS) measurements of Glx and glutamate in the anterior cingulate cortex (ACC) and thalamus in patients with first episode psychosis (*n* = 23) at clinical presentation, and after 6 weeks and 9 months of treatment with antipsychotic medication. At 9 months, patients were classified into Remission (*n* = 12) and Non-Remission (*n* = 11) subgroups. Healthy volunteers (*n* = 15) were scanned at the same three time-points. In the thalamus, Glx varied over time according to remission status (*P* = 0.020). This reflected an increase in Glx between 6 weeks and 9 months in the Non-Remission subgroup that was not evident in the Remission subgroup (*P* = 0.031). In addition, the change in Glx in the thalamus over the 9 months of treatment was positively correlated with the change in the severity of Positive and Negative Syndrome Scale (PANSS) positive, total and general symptoms (P<0.05). There were no significant effects of group or time on glutamate metabolites in the ACC, and no differences between either patient subgroup and healthy volunteers. These data suggest that the nature of the response to antipsychotic medication may be related to the pattern of changes in glutamatergic metabolite levels over the course of treatment. Specifically, longitudinal reductions in thalamic Glx levels following antipsychotic treatment are associated with symptomatic improvement.

## Introduction

In around one-third of patients with schizophrenia, treatment with antipsychotic medication is ineffective,^1–3^ but the underlying neurobiological mechanisms of treatment response are not well understood. Schizophrenia is associated with disruptions in brain glutamatergic neurotransmission,^4,5^ and recent neuroimaging studies have indicated that the nature of the antipsychotic response may be related to brain glutamate levels.^6–11^ In patients with first episode psychosis prior to treatment, elevated glutamate in the anterior cingulate cortex (ACC) have been associated with a lower likelihood of reaching remission after 4 weeks of amisulpride.^8^ Similarly, in established schizophrenia, higher levels of Glx (the combined signal from glutamate plus glutamine) in the medial frontal cortex have been associated with a poor response after restarting antipsychotic medication.^10^ Elevated ACC glutamatergic metabolites have also been reported in first episode patients who had failed to achieve remission following antipsychotic treatment,^7^ in patients who were treatment resistant^6,9^ and in patients resistant to clozapine.^12^ Elevated glutamate metabolites in treatment-resistant schizophrenia have also been described in the caudate nucleus.^11^

While brain glutamate metabolite levels have thus been related to antipsychotic response,^8^ levels of these metabolites may be reduced by antipsychotic medication.^13^ In patients with first episode psychosis, longitudinal reductions in glutamate in the ACC and left thalamus have been observed over 4 weeks of antipsychotic treatment,^8^ and longitudinal reductions in glutamine and Glx in the left thalamus have been reported after 30 and 80 months of treatment.^14,15^ Glutamate reductions have also been reported in the frontal cortex, following 4 and 6 months of antipsychotic treatment,^16–18^ and in the striatum following 4 weeks of antipsychotic treatment.^18,19^ However, reductions in glutamatergic metabolites in the thalamus^20^ or ACC^20,21^ have not been detected by other studies. Studies in patients with chronic schizophrenia have produced mixed findings, some reporting reductions in glutamate levels following antipsychotic treatment in the frontal^22^ and temporal cortex,^23^ but others finding no change (in frontal cortex,^23–25^ temporal cortex^25^ and thalamus^23,25^).

If there are longitudinal reductions in glutamate levels with antipsychotic treatment, we hypothesised that these may be related to symptomatic improvement. The present study aimed to test this by examining the relationship between glutamate metabolites in the ACC and thalamus and remission status at three timepoints over the first 9 months of antipsychotic treatment in patients with first episode psychosis. The present dataset is an extension to our previous study, which investigated the relationship between glutamate and treatment response over 4 weeks, reporting that elevated glutamate in the ACC at first presentation predicted poor antipsychotic response.^8^ Here we extend to a longer follow-up period of 9 months in a subset of this cohort. This duration of treatment corresponds to that in our previous cross-sectional study, which found that after 9 months of treatment, first episode patients who had not achieved remission had higher ACC glutamate levels than those in remission.^7^ To aid interpretation of our findings, we also assessed a sample of healthy volunteers over the same time period.

## Results

At 9 months, 12 patients met Remission criteria and 11 patients met criteria for Non-Remission. There were no significant differences in demographic variables between the Remission and Non-Remission subgroups, in substance use (Supplementary Table 1) or in duration of or adherence to antipsychotic medication at any timepoint (Table 1 and Supplementary Notes). At the time of the baseline scan, 4 patients were medication naïve, and all but one of the remaining patients were receiving amisulpride. At the 6 weeks and 9 month timepoint the Remission and Non-Remission groups were taking a similar set of antipsychotic drugs, and did not differ in chlorpromazine equivalent dose (Table 1). Please see Table 1 and Supplementary Notes for group differences in PANSS scores at each timepoint.

**Table 1:** Subject demographics and clinical characteristics

	Healthy Volunteers *n* = 15	Total FEP Patient Group *n* = 23	Non-Remission *n* = 11	Remission *n* = 12
*Baseline*				
Age (years)	24.5 (4.5)	25.5 (5.1)	25.6 (5.3)	25.4 (5.2)
Male/Female	12/3	17/6	10/1	7/5
Education, years	14.5 (2.7)	11.5 (3.1)**	11.6 (2.3)	11.5 (3.7)
Currently employed Y/N	14/1	12/11**	4/7	8/4
Ethnicity (White/Black/Asian/Other)	8/3/2/2	8/8/2/5	5/3/1/2	3/5/1/3
Duration of psychosis (months)		13.3 (11.2)	17.0 (9.0)	9.9 (12.3)
Duration of treatment (days)		10 (9)	11 (11)	10 (7)
Antipsychotics: None/Amisulpride/Risperidone		4/18/1	2/9/0	2/9/1
CPZ Equivalent Dose		117.1 (77.2)	125.1 (78.0)	109.8 (79.2)
PANSS Positive		19.3 (4.7)	20.8 (5.4)	17.9 (3.6)
PANSS Negative		14.5 (5.4)	16.8 (6.3)	12.3 (3.6)***
PANSS General		34.4 (8.5)	36.4 (9.0)	32.6 (8.0)
PANSS Total		68.2 (15.9)	74.0 (17.5)	62.8 (12.6)
PSP		54.6 (10.4)	54.1 (8.5)	55.1 (12.2)
*6 Weeks*				
Duration of treatment (days)		42.6 (14.4)	37.7 (14.9)	47.1 (13.0)
None/Amisulpride/Risperidone/Olanzapine/Quetiapine/Aripiprazole		1/16/1/1/2/2	1/8/0/1/1/0	0/8/1/0/1/2
CPZ Equivalent Dose		213.7 (109.1)	188.5 (89.9)	236.9 (123.5)
Adherence (% days on medication)		90.8 (22.8)	94.0 (14.9)	87.8 (28.7)
PANSS Positive		13.0 (5.3)	15.5 (5.8)	10.7 (3.5)*
PANSS Negative		13.2 (6.0)	16.7 (6.8)	9.9 (2.1)**
PANSS General		27.0 (8.5)	30.0 (9.2)	24.3 (7.2)
PANSS Total		53.2 (18.2)	62.3 (20.2)	44.9 (11.5)*
PSP		64.7 (16.2)	57.6 (12.9)	71.1 (16.8)*
Weeks between 1st and 2nd scan [Mean (SD Range)]	16 (28, 4–86)	7 (12, 4–63)	4 (0.5, 4–5)	10 (17, 4–63)
*9 Months*				
Duration of treatment (days)		200.0 (155.2)	189.8 (140.0)	209.4 (173.6)
None/Amisulpride/Risperidone/Olanzapine/Quetiapine/Aripiprazole		8/6/1/5/1/2	4/2/0/4/1/0	4/4/1/1/0/2
CPZ Equivalent Dose		234.7 (86.1)	236.0 (64.9)	233.6 (104.8)
Adherence (% days on medication)		73.6 (30.8)	72.8 (30.7)	74.3 (32.3)
PANSS Positive		13.4 (6.0)	17.6 (5.3)	9.6 (3.4)***
PANSS Negative		11.3 (3.9)	13.1 (4.0)	9.6 (3.1)***
PANSS General		28.4 (7.1)	32.6 (6.0)	24.5 (5.8)***
PANSS Total		53.1 (14.4)	63.4 (11.8)	43.7 (9.2)***
PSP		56.5 (18.1)	48.8 (14.3)	63.6 (18.8)*
Months between 1st and 3rd scan [Mean (SD Range)]	8 (7, 2–21)	9 (6, 2–19)	8 (5, 2–17)	9 (6, 2–19)

Mean and (Standard Deviation) presented. Remission and Non-Remission groups were classified based on presentation at the 9 month timepoint

Significant differences between the Remission and Non-Remission group are denoted in the Remission group column

Significant differences between Healthy Volunteers and FEP patients are denoted by *P <0.05, **P < 0.01, ***P < 0.001 in the FEP patient column

*FEP* first episode psychosis, *PANSS* positive and negative syndrome scale, *PSP* personal and social performance scale, *CPZ* *equivalent dose* chlorpromazine equivalent dose

Variables relating to 1H-MRS data quality are provided in Supplementary Table 2. For one patient Glx and glutamate data from the thalamus were below 20% CRLB, reducing the sample to *n* = 22, and for one healthy volunteer Glx data from the thalamus were below 20% CRLB, reducing the sample to *n* = 14. There were no significant group differences for spectra quality (Supplementary Table 2) or voxel tissue content (Table 2).

**Table 2:** 1H-MRS metabolite concentrations corrected for voxel cerebrospinal fluid (CSF) content, and 1H-MRS voxel % of white matter, grey matter and CSF, at three timepoints

	Healthy volunteers *n* = 15	Total patient group *n* = 23	Non-Remission *n* = 11	Remission *n* = 12
*Baseline 1H-MRS Scan*				
Anterior cingulate cortex				
Glx	20.76 (2.96)	19.99 (3.29)	19.85 (3.48)	20.12 (3.27)
Glu	14.04 (1.36)	14.11 (1.61)	13.87 (1.23)	14.33 (1.92)
NAA	12.24 (1.19)	11.97 (1.35)	11.98 (1.30)	11.97 (1.46)
Cho	2.77 (0.51)	2.80 (0.34)	2.83 (0.46)	2.76 (0.19)
mI	7.95 (1.62)	7.75 (1.28)	7.77 (1.25)	7.74 (1.37)
Cr	10.29 (1.24)	10.46 (1.17)	10.34 (1.29)	10.57 (1.10)
White matter	12.54 (0.04)	12.36 (0.04)	13.19 (0.04)	11.61 (0.03)
Grey matter	66.43 (0.06)	65.00 (0.04)	64.95 (0.05)	65.04 (0.04)
CSF	20.99 (0.04)	22.63 (0.05)	21.83 (0.05)	23.35 (0.05)
Left Thalamus				
Glx	11.04 (2.53)	10.30 (2.10)	10.04 (1.62)	10.54 (2.52)
Glu	8.13 (1.01)	8.06 (1.33)	7.96 (0.61)	8.15 (1.78)
NAA	11.31 (1.29)	11.57 (0.72)	11.73 (0.84)	11.43 (0.61)
Cho	2.18 (0.29)	2.08 (0.18)	2.14 (0.17)	2.02 (0.18)
mI	4.21 (0.82)	4.08 (0.55)	4.16 (0.39)	4.01 (0.68)
Cr	7.37 (0.64)	7.53 (0.59)	7.48 (0.54)	7.57 (0.65)
White matter	73.94 (0.08)	77.09 (0.06)	79.19 (0.06)	75.17 (0.06)
Grey matter	25.67 (0.08)	22.51 (0.06)	20.24 (0.05)	24.57 (0.06)
CSF	00.37 (0.01)	00.40 (0.01)	00.56 (0.01)	00.25 (0.00)
*6 weeks 1H-MRS Scan*				
Anterior cingulate cortex				
Glx	19.70 (3.46)	20.34 (2.95)	20.02 (2.68)	20.63 (3.26)
Glu	13.18 (1.74)	14.13 (2.01)	14.07 (2.18)	14.18 (1.94)
NAA	12.43 (1.22)	12.43 (1.28)	12.36 (0.90)	12.49 (1.59)
Cho	2.73 (0.44)	2.90 (0.44)	2.97 (0.24)	2.83 (0.57)
mI	8.09 (1.41)	8.17 (1.42)	8.22 (1.16)	8.12 (1.67)
Cr	10.42 (1.06)	10.88 (1.27)	10.85 (0.72)	10.90 (1.65)
White matter	10.53 (0.03)	11.06 (0.02)	11.23 (0.02)	10.91 (0.03)
Grey matter	66.64 (0.04)	65.55 (0.05)	65.34 (0.04)	65.74 (0.05)
CSF	22.90 (0.05)	23.37 (0.05)	23.40 (0.05)	23.34 (0.06)
Left Thalamus				
Glx	9.63 (2.61)	9.65 (1.77)	9.56 (1.61)	9.73 (1.98)
Glu	7.57 (1.79)	7.26 (1.07)	7.28 (0.90)	7.23 (1.24)
NAA	11.71 (0.69)	11.26 (0.71)	11.26 (0.70)	11.25 (0.76)
Cho	**2.22 (0.23)**	2.06 (0.17)*	2.10 (0.16)	2.02 (0.18)
mI	4.28 (0.64)	4.10 (0.78)	4.14 (0.97)	4.05 (0.61)
Cr	7.55 (0.68)	7.38 (0.48)	7.43 (0.39)	7.34 (0.56)
White matter	77.47 (0.09)	80.30 (0.05)	79.84 (0.05)	80.72 (0.05)
Grey matter	22.34 (0.09)	19.49 (0.05)	19.91 (0.05)	19.10 (0.05)
CSF	00.25 (0.00)	00.19 (0.00)	00.22 (0.00)	00.15 (0.00)
*9 month 1H-MRS Scan*				
Anterior cingulate cortex				
Glx	20.58 (3.32)	20.13 (2.26)	20.45 (2.60)	19.83 (1.97)
Glu	14.19 (2.43)	13.65 (2.03)	13.61 (1.93)	13.68 (2.19)
NAA	12.24 (1.23)	12.05 (1.21)	12.16 (1.21)	11.95 (1.26)
Cho	2.76 (0.35)	2.73 (0.29)	2.75 (0.38)	2.71 (0.19)
mI	7.47 (1.15)	7.88 (1.02)	8.13 (1.22)	7.65 (0.78)
Cr	10.34 (1.31)	10.33 (0.68)	10.26 (0.73)	10.40 (0.64)
White matter	10.43 (0.03)	12.98 (0.04)	13.35 (0.04)	12.64 (0.03)
Grey matter	66.93 (0.04)	64.58 (0.05)	63.61 (0.06)	65.45 (0.04)
CSF	22.61 (0.04)	22.42 (0.04)	23.02 (0.04)	21.88 (0.04)
Left Thalamus				
Glx	10.14 (1.92)	10.60 (1.93)	**11.46 (1.45)**	9.75 (2.02)*
Glu	7.70 (1.43)	8.31 (1.24)	8.60 (0.89)	8.03 (1.51)
NAA	11.05 (0.96)	11.54 (1.46)	11.48 (1.71)	11.61 (1.26)
Cho	2.05 (0.39)	2.12 (0.36)	2.21 (0.45)	2.05 (0.24)
mI	4.17 (0.79)	4.42 (0.78)	4.55 (0.61)	4.32 (0.91)
Cr	7.23 (0.59)	7.60 (1.04)	7.70 (1.39)	7.50 (0.61)
White matter	77.80 (0.09)	76.26 (0.07)	75.75 (0.06)	76.72 (0.07)
Grey matter	21.83 (0.08)	23.42 (0.06)	23.94 (0.05)	22.95 (0.07)
CSF	00.37 (0.01)	00.30 (0.00)	00.28 (0.00)	00.31 (0.00)

Data are presented as mean (SD)

Significant group differences are represented by *P < 0.05

*Glu* Glutamate, *NAA* N-acetyl-aspartate, *Cr* creatine, *mI* myo-inositol, *Cho* choline

**Fig. 1:**
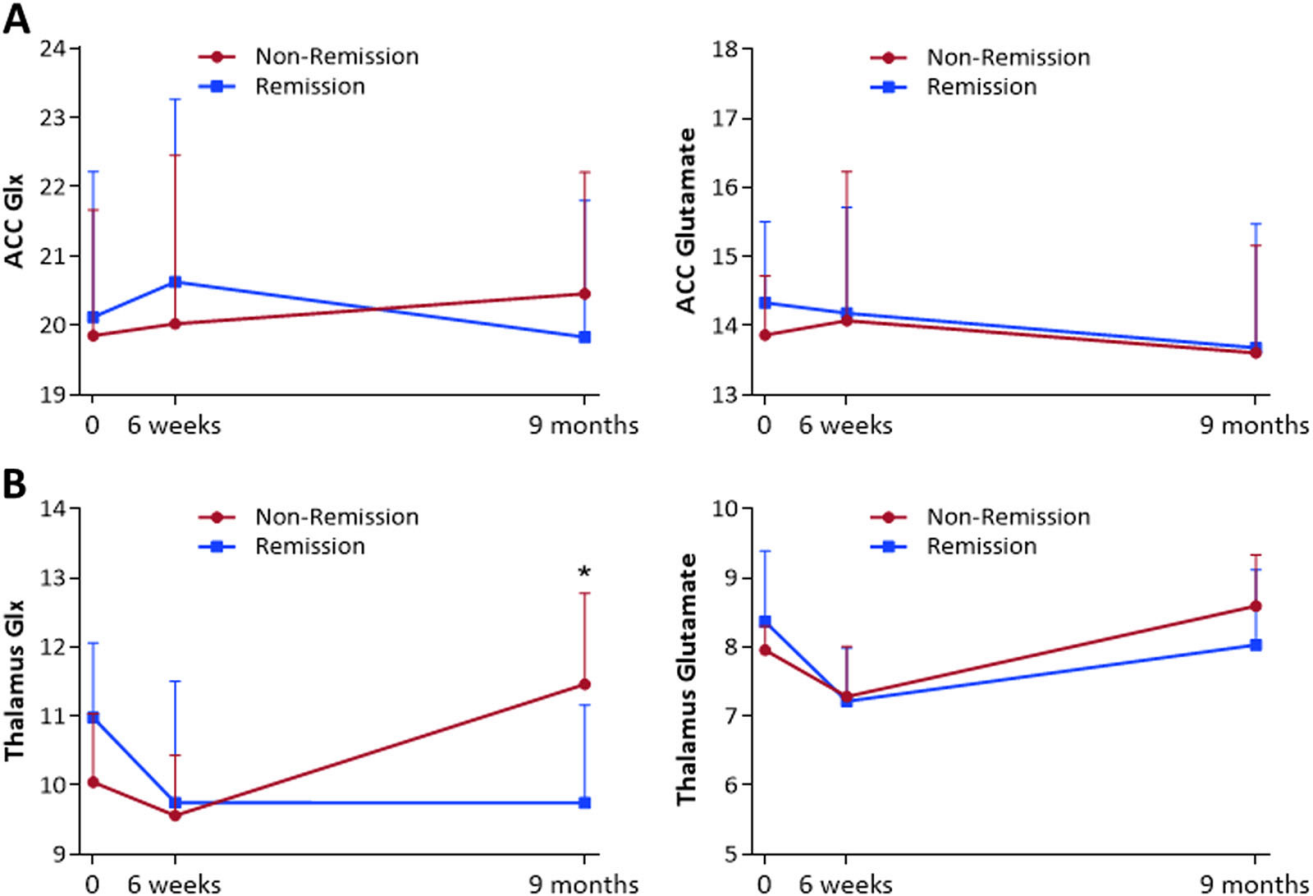
Glx (left) and Glutamate (right) at Baseline, 6 weeks and 9 months, in Remission and Non-Remission groups in (**a**) anterior cingulate cortex and (**b**) left thalamus *Represents higher thalamic Glx levels in the Non-Remission group compared to the Remission group at 9 months (*P* = 0.033). Glx and glutamate values are CSF-corrected, presented as mean & within-subjects standard deviation

**Fig. 2:**
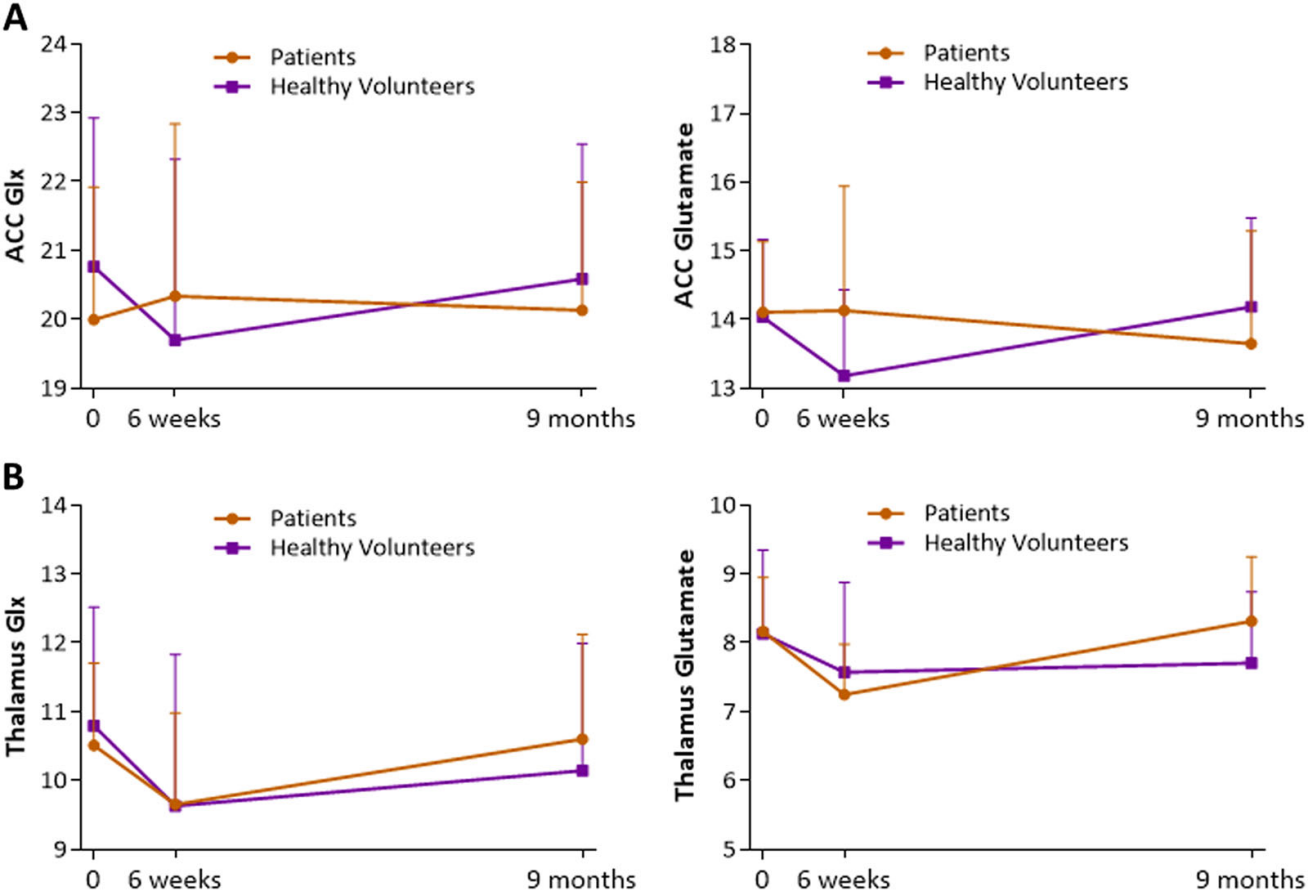
Glx (left) and Glutamate (right) at Baseline, 6 weeks and 9 months, in First Episode Psychosis (FEP) patients and Healthy Volunteers in (**a**) anterior cingulate cortex and (**b**) left thalamus

For both Glx and Glutamate in the ACC, there were no significant main or interaction effects of remission status or time (Fig. 1, Supplementary Table 3). This was also the case when time to follow-up was included as a covariate, and when analysis was restricted to patients who were adherent to antipsychotic medication at least 75% of the time. There were no significant relationships between the longitudinal percentage change in ACC glutamatergic metabolites and the percentage change in symptoms over time (Supplementary Table 4). There was also no significant difference in ACC glutamate metabolite levels over time in healthy volunteers compared to the overall patient sample (Fig. 2).

Glx levels in the left thalamus showed a significant interaction between remission status and time (F(2,40) = 4.337, *P* = 0.020, repeated measures ANOVA, Fig. 1). The main effects of remission status (F(1,20) = 0.121, *P* = 0.731) and time (F(2,40) = 2.541, *P* = 0.091) were non-significant. At 9 months, Glx levels in the thalamus were significantly higher in the Non-Remission compared to Remission group (F(1,20) = 5.244, *P* = 0.033, Cohen’s *d* = 0.98, one-way ANOVA). This was related to a significant effect of time in the Non-Remission group (F(2,20) = 6.183, *P* = 0.008, repeated measures ANOVA), which reflected an increase in Glx concentration between 6 weeks and 9 months (*P* = 0.031, Cohen’s *d* = 1.24, Bonferroni-corrected pairwise comparisons). Within the Remission subgroup, Glx levels did not vary significantly over time (F(2,20) = 1.849, *P* = 0.183, repeated measures ANOVA). Similar results were obtained when the analysis was restricted to patients who reported being medication adherent at least 75% of the time (Supplementary Figure 2). There were no significant differences in thalamic Glx levels over time in the healthy volunteer group compared to the overall patient sample (Fig. 2, Supplementary Table 3).

Glutamate levels in the left thalamus showed a significant effect of time (F(2,40) = 7.306, *P* = 0.002, repeated measures ANOVA), while the main effects of remission status (F(1,20) = 0.036, *P* = 0.852) and the remission status x time interaction were not significant (F(2,40) = 1.310, *P* = 0.281, Fig. 1). The effect of time reflected a significant decrease in thalamic glutamate across both patient subgroups between baseline and 6 weeks (*P* = 0.005, Bonferroni-corrected pairwise comparisons), and a significant increase between 6 weeks and 9 months (*P* = 0.010, Cohen’s *d* = −0.67). The results remained the same when the analysis was restricted to patients who were adherent to antipsychotic medication at least 75% of the time. When the entire patient sample was compared to the healthy volunteer sample, the effect of time on glutamate in the thalamus was apparent across all subjects (healthy volunteers and patients) (F(2,70) = 3.753, *P* = 0.028, repeated measures ANOVA), and was related to a significant decrease in glutamate between baseline and 6 weeks (*P* = 0.045, Cohen’s *d* = −0.52, Bonferroni-corrected pairwise comparisons, Fig. 2). No significant effect of diagnostic group, and no interaction were found (Supplementary Table 3).

**Fig. 3:**
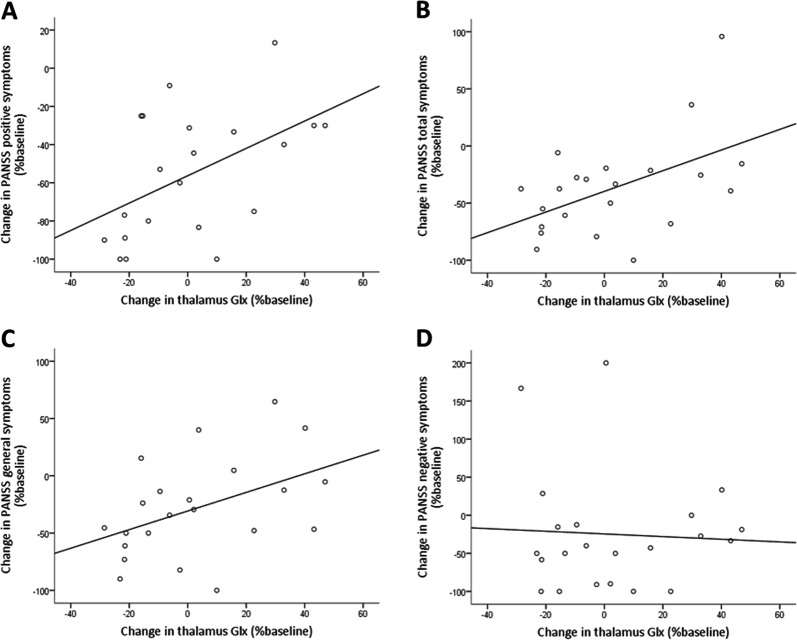
Correlations between change in PANSS score (−100% indicates full symptomatic improvement, whereas 0% denotes no change in symptoms) and change in thalamic Glx levels over 9 months (negative values indicate reduction in thalamic Glx levels, whereas positive values indicate increase in thalamic Glx). **a** Significant positive correlation between the percentage change in Glx levels in the thalamus and the percentage change in PANSS positive score (*r* = .493, *P* = 0.023), (**b**) PANSS total score (*r* = .501, *P* = 0.018) and (**c**) PANSS general score (*r* = .446, *P* = 0.037), between baseline and 9 months. **d** No significant correlation for the percentage change in PANSS negative score

There was a positive correlation between the percentage change in Glx levels in the thalamus and the percentage change in PANSS positive score between baseline and 9 months (*r* = .512, *P* = 0.015, Pearson’s bivariate correlation): the greater the longitudinal reduction in thalamic Glx, the greater the improvement in positive symptoms over the course of treatment (decrease in PANSS positive score). This correlation remained significant when one outlying value identified using Cook’s D was excluded (*r* = .493, *P* = 0.023, Fig. 3). Secondary analyses found positive correlations between the percentage change in Glx in the thalamus and the percentage change in PANSS general (*r* = .446, *P* = 0.037) and PANSS total (*r* = .501, *P* = 0.018) scores, but not the PANSS negative score (*r* = -.053, *P* = 0.815, Fig. 3) or PSP score (*r* = -.135, *P* = 0.550). Relationships remained significant when partial correlations were conducted to control for time to follow-up.

In contrast, there were no significant relationships between the percentage change in glutamate in the thalamus and percentage symptom change (Supplementary Table 4).

Repeated measures MANOVA analyses assessed metabolite changes over time for N-acetyl-aspartate, creatine, myo-inositol, and choline (Supplementary Table 5). There were no significant main effects of group, time or interaction in remission versus non-remission groups in the ACC or left thalamus, or in patients versus healthy volunteers in the ACC. In the thalamus, there was a significant interaction between group (patient vs healthy volunteer) and time (F(2,70) = 3.520, *P* = 0.010). Post-hoc tests did not find significant effects when groups and timepoints were analysed separately.

## Discussion

This study investigated the relationship between brain glutamatergic metabolites and the response to antipsychotic medication over the first 9 months of treatment for psychosis. The main finding was that Glx in the thalamus increased over time in Non-Remitters, such that after 9 months Glx levels were higher in patients who were not in remission than in those who were. Furthermore, symptomatic improvement over the course of treatment was associated with a longitudinal reduction in thalamic Glx levels. These results extend our previous observations over shorter periods of treatment^8^ to indicate that longer-term symptomatic response may be linked to the level of glutamatergic metabolites.

In a recent longitudinal study over 4 weeks of antipsychotic treatment (containing an overlapping sample of the participants to the current study) we also found that glutamate levels decreased over time in the thalamus, but there was no significant relationship between the reduction over this timeframe and symptomatic improvement.^8^ In this extended study, the treatment period was 9 months, which suggests that longitudinal differences in relation to symptomatic response may emerge after longer periods of treatment. This 9 month period is comparable to the time since presentation in an earlier cross-sectional study in first episode psychosis, in which we also observed numerically but non-significantly higher thalamic Glx in the Non-Remission compared to Remission group.^7^

The results of the present study are broadly consistent with a previous report in patients with schizophrenia showing that higher social and occupational functioning scores 80 months after diagnosis are associated with a greater degree of thalamic Glx reduction over those 80 months.^14^ Together these findings suggest that thalamic Glx levels may be more related to symptomatic outcome after a period of several months, rather than the initial period of treatment. A recent study observed a trend for an increase in thalamic Glx levels over 5 years in first episode psychosis patients, although the relationship with treatment response was not investigated.^26^ At 9 months, there were no group differences in substance use or spectral quality. However, there was a numerically higher percentage of cannabis users in the non-remission group, with a higher frequency of use. It is possible that cannabis use or other unknown external factors may have contributed to the observed increase in thalamic Glx in non-remitters.^27^

Thalamocortical dysconnectivity is thought to be a key pathophysiological feature of schizophrenia,^28^ and may be mediated by alterations in thalamic glutamatergic transmission.^29,30^ Human neuroimaging studies have demonstrated that antipsychotic administration can modify thalamic activity and metabolism,^31–34^ but there are fewer data on the role of the thalamus and its cortical connectivity in mediating clinical outcome, with some studies^35–37^ but not others^34,38^ suggesting an association. This could be explored in future work combining serial 1H-MRS glutamate and functional connectivity measurements in relation to early and longer-term clinical outcomes.

Contrary to our expectations, we did not detect any significant relationships between remission status and glutamatergic metabolite levels in the ACC. This is inconsistent with most previous reports linking antipsychotic non-response to elevated glutamate in the ACC,^6–10,12^ although one other study did not detect differences in ACC glutamate in relation to response.^11^ In our recent study that involved a larger sample (*n* = 46) overlapping with the present cohort,^8^ non-remission at 4 weeks was associated with elevated ACC glutamate prior to treatment with amisulpride. The lack of significant difference in the current smaller sample (*n* = 23) may reflect limitations of sample size. In addition, compared to our larger study,^8^ a greater proportion of participants in the present study had received antipsychotics prior to baseline, which may have affected ACC glutamate metabolite levels.^13^

In line with data from previous studies,^5,8,21^ we did not detect significant differences in thalamic or ACC Glx or glutamate concentrations between the total patient sample and healthy volunteers. Although a recent meta-analysis of the literature suggests that there may be differences between first episode patients and healthy volunteers in glutamine levels in the thalamus and ACC,^5^ the acquisition parameters we used at 3 Tesla did not allow reliable quantification of glutamine. While Glx in the thalamus increased over time in Non-Remitters, this effect did not reach significance for the glutamate signal alone. This may relate to differences in the Glx versus glutamate measurement, or could indicate that glutamine is contributing to this effect.

A strength of this study is the relatively long follow-up period of 9 months in comparison to most previous studies^16,19,22,23,25,39^ which, together with scanning early in treatment permitted investigation of the relationships between brain glutamate and short and longer-term outcome under antipsychotic treatment. A further strength is sample homogeneity, through the inclusion of participants in their first episode of psychosis who had received minimal prior antipsychotic medication.

One limitation of the study was that the majority of participants were not antipsychotic naïve at baseline and may have already experienced initial symptomatic improvement. Even short-term antipsychotic exposure may reduce glutamate levels^8^ and may have reduced our ability to detect subsequent reductions. The 52% response rate in the present sample is slightly lower than that reported in the literature in first episode psychosis (approximately 60%^40–43^), which may be accounted for by prior medication exposure, or by the longer follow-up time period. Other limitations include controlling for the potential effects of medication adherence, which was estimated through self-report and clinical notes. Although the findings remained significant when the analysis was restricted to participants who reported being medication adherent at least 75% of the time, inclusion of more accurate measures of adherence, such as antipsychotic plasma levels, would have been helpful. The majority of patients initially received the same antipsychotic medication, amisulpride, which is a relatively selective D2 dopamine receptor antagonist.^44^ However, subsequently there was more variation in the particular antipsychotic medications used. Differences in the pharmacological profile of antipsychotics could have differential effects on glutamatergic neurotransmission,^45^ which may have increased variability over the observation period. Nevertheless, at the 9 month timepoint, the Remission and Non-Remission groups were taking a similar set of antipsychotic drugs, the levels of medication adherence were comparable, and a similar proportion of patients were no longer taking medication. The data also showed a reduction in thalamic glutamate across all participants between baseline and 6 weeks, which may reflect a methodological factor impacting on the measurement. This indicates the utility of including a healthy volunteer or other non-intervention group for interpretation of longitudinal studies. Lastly, this study used adapted Andreasen’s criteria for remission, consistent with previous studies.^7,40^ Therefore our study did not account for fluctuations in symptoms or remission status that may have occurred over the 9 month period, which would require regular symptom monitoring.

In summary, the findings of the present study extend the literature linking ACC glutamate to antipsychotic response^6–10^ by indicating that response to antipsychotic medication over the first 9 months of treatment may be related to longitudinal changes in glutamatergic metabolites in the thalamus. The association between elevated thalamic Glx levels and Non-Remission is consistent with the notion that brain glutamate transmission is a potential therapeutic target for novel treatments for psychosis.

## Methods

The study included participants recruited in two studies: OPTiMiSE (Optimisation of Treatment and Management of Schizophrenia in Europe; www.optimisetrial.eu; EudraCT-Number: 2010-020185-19; clinicaltrials.gov identifier: NCT01248195^40^) (*n* = 34, using the London sample from the OPTiMiSE study^8^), and TRFEP (The neurobiological determinants of treatment response in psychosis^46^; reference number 12/EE/0220) (*n* = 6, with an additional *n* = 3 taking part in both studies). Both studies were granted ethical approval by the South London and Maudsley NHS Trust Ethics Committee, and all participants provided written informed consent. Patients were recruited from early intervention community teams and wards. We aimed to recruit 24 participants to detect a change in Glx levels with antipsychotic treatment, according to power calculations reported in a recent meta-analysis.^13^ Of 43 patients who agreed to participate in the study, a total of *n* = 23 patients completed all 3 scans (*n* = 14 from the OPTiMiSE study, *n* = 6 from the TRFEP study, and *n* = 3 taking part in both studies) (Supplementary Fig. 1). In the patient group, inclusion required presentation with a first episode of psychosis within the past 2 years, aged between 18–40, and a diagnosis of a psychotic disorder according to ICD 10 criteria or DSM-IV criteria. Inclusion required previous antipsychotic exposure of <15 days (OPTiMiSE study), or no exposure to antipsychotic medication within the past 6 weeks (TRFEP study). Exclusion criteria included being unable to provide written informed consent, being coercively treated or being under legal custody. Healthy volunteers (*n* = 36) were recruited through online advertisements, with *n* = 15 completing all three MRI sessions. Healthy volunteers were 18–40 years old with no history of psychiatric illness. All subjects had no history of head injury or contraindications to MRI scanning.

In the patient sample, symptoms were assessed using the Positive and Negative Syndrome Scale (PANSS),^47^ and functioning was assessed using the Personal and Social Performance (PSP) scale at each MRI scan visit. Medication adherence and illicit drug use was determined using clinical notes and self-report of dates when medication was taken. Chlorpromazine Equivalent Doses were calculated.^48^ The primary clinical outcome measure was remission at 9 months, based upon adapted Andreasen criteria,^49^ consistent with our larger study,^40^ and previous cross-sectional study in first episode psychosis.^7^

MRI scans were conducted at baseline and repeated after a mean of 6 weeks and 9 months. All data were acquired at 3-Tesla on a General Electric Healthcare (Chicago, USA) HDxt MR system. The same sequences were acquired at each time-point. Whole brain sagittal T1-weighted images were acquired using a modified ADNI GO protocol (See http://adni.loni.usc.edu/methods/documents/mri-protocols/) with an echo time (TE) 2.848 ms; repetition time(TR) 6.984 ms; inversion time 400 ms; flip angle 11°, Field of view 260 mm, slice thickness 1.2 mm, matrix size 256x256 mm. The structural images were reformatted to axial orientation for 1H-MRS voxel positioning in the bilateral ACC and left thalamus. The centre of the ACC voxel (20 x 20 x 20 mm) was positioned 16 mm superior to the anterior portion of the genu of the corpus callosum on the midline sagittal localiser, avoiding the corpus callosum. The voxel in the left thalamus (15 x 20 x 20 mm) was also positioned from the axial image, using the coronal and sagittal localisers to minimise cerebrospinal fluid (CSF) content in the voxel (voxel placement and example spectra previously published^8^).

1H-MRS spectra were acquired using PRESS (Point RESolved Spectroscopy), at TE = 30 msec; TR = 3000 msec; 96 averages; bandwidth/sample frequency = +/- 2500Hz; number of complex points = 4096. Data were acquired using the standard GE PROBE (PROton Brain Examination) sequence, which includes acquisition of unsuppressed water reference spectra (16 averages). The target water line-widths after shimming were < 7Hz in the ACC and < 10Hz in the left thalamus. For follow-up scans, radiographers referred to the baseline scan voxel position to reduce variability in voxel placement.

Spectra were analyzed using LC Model version 6.3-0I^50,51^ using a standard LC Model basis set acquired using PRESS at 3-Tesla and a TE of 30msec containing 16 metabolites. Poorly fitted metabolite peaks (Cramer–Rao lower variance bounds (CRLB) >20% as reported by LCModel) were excluded from further analysis. All metabolite values are reported in institutional units.

To correct metabolite concentration estimates for voxel CSF content, T1-weighted images were segmented into grey matter, white matter and CSF images using Statistical Parametric Mapping 8, version 6313 (SPM8; Wellcome Department of Imaging Neurosciences, University College London, UK). Voxel coordinates were obtained from spectra file headers using General Electric’s spectroscopy processing tool SAGE and mapped against the T1-weighted structural images using in-house software, to calculate the percentage tissue content of the individual 1H-MRS voxels. Metabolite values were then corrected using the following equation^51^:

$$\begin{array}{l}{\rm{Uncorrected}}\,{\rm{metabolite}} \times \left( {{\rm{wm}} + 1.21 \times {\rm{gm}} + 1.55 \times {\rm{csf}}} \right) / \left( {{\rm{gm}} + {\rm{wm}}} \right)\\ {\rm{gm}} = {\rm{grey}}\,{\rm{matter}}\\ {\rm{wm}} = {\rm{white}}\,{\rm{matter}}\\ {\rm{csf}} = {\rm{cerebrospinal}}\,{\rm{fluid}}\end{array}$$

Statistical analyses were performed using SPSS version 23 (SPSS inc. Chicago, IL, USA). For demographic and clinical data, between group differences were assessed using Fisher’s Exact Test (2 tailed) and independent samples Student’s t-test. Equal variances were assumed unless Levene’s test was significant.

The main 1H-MRS metabolites of interest were Glx and glutamate, corrected for voxel CSF content. Repeated measures ANOVA assessed the effects of time, group and group*time on voxel Glx and glutamate levels. A significant effect of time was followed up with Bonferroni-corrected pairwise comparisons (to determine significant differences between timepoints). A significant effect of group was followed up by one-way ANOVA tests (to determine group differences at separate timepoints). A significant interaction was followed up with one-way ANOVA tests, and also a repeated measures ANOVA in the remission and in the non-remission groups separately, with Bonferroni-corrected pairwise comparisons (to determine significant differences between timepoints in each group seperately). The primary analysis compared the Remission and Non-Remission patient groups. Subsequent analyses compared the healthy volunteer group to the total patient group. Relationships between the percentage change in PANSS score (minus minimum possible scores)^52^ or PSP score, and the percentage change in Glx and glutamate over 9 months were assessed using Pearson’s bivariate correlations (2 tailed). Outliers were identified using Cook’s distance estimates, excluding values higher than 4/n. Repeated measures MANOVA assessed metabolite changes over time for other metabolites. The data that support the findings of this study are available from the corresponding author upon reasonable request.

### Reporting summary

Further information on research design is available in the Nature Research Reporting Summary linked to this article.

## Supplementary information

Supplementary Information

Reporting Summary

## Data Availability

The data that support the findings of this study are available from the corresponding author upon reasonable request.

## Supplementary information

**Supplementary information** accompanies the paper on the *npj Schizophrenia* website (10.1038/s41537-019-0080-1).

## References

[1] Lehman AF (2004). Practice guideline for the treatment of patients with schizophrenia, second edition. Am. J. Psychiatry.

[2] Demjaha A (2017). Antipsychotic treatment resistance in first-episode psychosis: prevalence, subtypes and predictors. Psychol. Med..

[3] Lally J, Gaughran F, Timms P, Curran S (2016). Treatment-resistant schizophrenia: current insights on the pharmacogenomics of antipsychotics. Pharmgenomics. Pers. Med..

[4] Ripke S (2014). Biological insights from 108 schizophrenia-associated genetic loci. Nature.

[5] Merritt, K., Egerton, A., Kempton, M. J., Taylor, M. J. & McGuire, P. K. Nature of glutamate alterations in schizophrenia a meta-analysis of proton magnetic resonance spectroscopy studies. *JAMA Psychiatry* **73**, 665–674 (2016). 10.1001/jamapsychiatry.2016.0442 27304221

[6] Demjaha A (2014). Antipsychotic treatment resistance in schizophrenia associated with elevated glutamate levels but normal dopamine function. Biol. Psychiatry.

[7] Egerton A (2012). Anterior cingulate glutamate levels related to clinical status following treatment in first-episode schizophrenia. Neuropsychopharmacology.

[8] Egerton A., Broberg B. V., Van Haren N., Merritt K., Barker G. J., Lythgoe D. J., Perez-Iglesias R., Baandrup L., Düring S. W., Sendt K. V., Stone J. M., Rostrup E., Sommer I. E., Glenthøj B., Kahn R. S., Dazzan P., McGuire P. (2018). Response to initial antipsychotic treatment in first episode psychosis is related to anterior cingulate glutamate levels: a multicentre 1H-MRS study (OPTiMiSE). Molecular Psychiatry.

[9] Mouchlianitis E (2016). Treatment-resistant schizophrenia patients show elevated anterior cingulate cortex glutamate compared to treatment-responsive. Schizophr. Bull..

[10] Szulc A (2013). Proton magnetic resonance spectroscopy measures related to short-term symptomatic outcome in chronic schizophrenia. Neurosci. Lett..

[11] Goldstein ME, Anderson VM, Pillai A, Kydd RR, Russell BR (2015). Glutamatergic neurometabolites in clozapine-responsive and -resistant schizophrenia. Int. J. Neuropsychopharmacol..

[12] Iwata Yusuke, Nakajima Shinichiro, Plitman Eric, Caravaggio Fernando, Kim Julia, Shah Parita, Mar Wanna, Chavez Sofia, De Luca Vincenzo, Mimura Masaru, Remington Gary, Gerretsen Philip, Graff-Guerrero Ariel (2019). Glutamatergic Neurometabolite Levels in Patients With Ultra-Treatment-Resistant Schizophrenia: A Cross-Sectional 3T Proton Magnetic Resonance Spectroscopy Study. Biological Psychiatry.

[13] Egerton, A. et al. Effects of antipsychotic administration on brain glutamate in schizophrenia: a systematic review of longitudinal^1^H-MRS studies. *Front. Psychiatry* **8**, 66 (2017). 10.3389/fpsyt.2017.00066 PMC5408014 28503156

[14] Aoyama N (2011). Grey matter and social functioning correlates of glutamatergic metabolite loss in schizophrenia. Br. J. Psychiatry.

[15] Theberge J (2007). Longitudinal grey-matter and glutamatergic losses in first-episode schizophrenia. Br. J. Psychiatry.

[16] Goto N (2012). Six-month treatment with atypical antipsychotic drugs decreased frontal-lobe levels of glutamate plus glutamine in early-stage first-episode schizophrenia. Neuropsychiatr. Dis. Treat..

[17] Stanley JA (1996). An in vivo proton magnetic resonance spectroscopy study of schizophrenia patients. Schizophr. Bull..

[18] de la Fuente-Sandoval C (2018). Prefrontal and striatal gamma-aminobutyric acid levels and the effect of antipsychotic treatment in first-episode psychosis patients. Biol. Psychiatry.

[19] de la Fuente-Sandoval C (2013). Glutamate levels in the associative striatum before and after 4 weeks of antipsychotic treatment in first-episode psychosis: a longitudinal proton magnetic resonance spectroscopy study. JAMA psychiatry.

[20] Bustillo JR (2010). 1H-MRS at 4 tesla in minimally treated early schizophrenia. Mol. Psychiatry.

[21] Kraguljac, N. V. et al. A longitudinal magnetic resonance spectroscopy study investigating effects of risperidone in the anterior cingulate cortex and hippocampus in schizophrenia. *Schizophr. Res*. 10.1016/j.schres.2018.12.028 (2019). 10.1016/j.schres.2018.12.028 PMC7881837 30630705

[22] Choe B, Suh T, Shinn K, Lee C, Paik I (1996). Observation of metabolic changes in chronic schizophrenia after neuroleptic treatment by in vivo hydrogen magnetic resonance spectroscopy. Invest. Radiol..

[23] Szulc A (2011). Proton magnetic resonance spectroscopy study of brain metabolite changes after antipsychotic treatment. Pharmacopsychiatry.

[24] Goff DC (2002). Modulation of brain and serum glutamatergic concentrations following a switch from conventional neuroleptics to olanzapine. Biol. Psychiatry.

[25] Szulc A (2005). The effect of risperidone on metabolite measures in the frontal lobe, temporal lobe, and thalamus in schizophrenic patients. A proton magnetic resonance spectroscopy (1H MRS). Pharmacopsychiatry.

[26] Galińska-Skok B (2019). Proton magnetic resonance spectroscopy changes in a longitudinal schizophrenia study: a pilot study in eleven patients. Neuropsychiatr. Dis. Treat..

[27] Schoeler T (2016). Continued versus discontinued cannabis use in patients with psychosis: a systematic review and meta-analysis. Lancet Psychiatry.

[28] Murray JD, Anticevic A (2017). Toward understanding thalamocortical dysfunction in schizophrenia through computational models of neural circuit dynamics. Schizophr. Res..

[29] Santana N, Troyano-Rodriguez E, Mengod G, Celada P, Artigas F (2011). Activation of thalamocortical networks by the n-methyl-d-aspartate receptor antagonist phencyclidine: reversal by clozapine. Biol. Psychiatry.

[30] Pratt J (2017). Thalamo-cortical communication, glutamatergic neurotransmission and neural oscillations: a unique window into the origins of ScZ?. Schizophr. Res..

[31] Lahti AC, Weiler MA, Medoff DR, Tamminga CA, Holcomb HH (2005). Functional effects of single dose first- and second-generation antipsychotic administration in subjects with schizophrenia. Psychiatry Res..

[32] Liddle PF, Lane CJ, Ngan ET (2000). Immediate effects of risperidone on cortico-striato-thalamic loops and the hippocampus. Br. J. Psychiatry.

[33] Holcomb HH (1996). Functional sites of neuroleptic drug action in the human brain: PET/FDG studies with and without haloperidol. Am. J. Psychiatry.

[34] Hadley JA (2014). Ventral tegmental area/midbrain functional connectivity and response to antipsychotic medication in schizophrenia. Neuropsychopharmacology.

[35] Bernard JA, Orr JM, Mittal VA (2017). Cerebello-thalamo-cortical networks predict positive symptom progression in individuals at ultra-high risk for psychosis. NeuroImage Clin..

[36] Rodríguez VM (1997). Fronto-striato-thalamic perfusion and clozapine response in treatment-refractory schizophrenic patients. A 99mTc-HMPAO study. Psychiatry Res..

[37] Molina Rodríguez V (1996). SPECT study of regional cerebral perfusion in neuroleptic-resistant schizophrenic patients who responded or did not respond to clozapine. Am. J. Psychiatry.

[38] Li F (2016). Longitudinal changes in resting-state cerebral activity in patients with first-episode schizophrenia: a 1-year follow-up functional mr imaging study. Radiology.

[39] Goff DC (2002). Modulation of brain and serum glutamatergic concentrations following a switch from conventional neuroleptics to olanzapine. Biol. Psychiatry.

[40] Kahn RS (2018). Amisulpride and olanzapine followed by open-label treatment with clozapine in first-episode schizophrenia and schizophreniform disorder (OPTiMiSE): a three-phase switching study. Lancet Psychiatry.

[41] Emsley RA (1999). Risperidone in the treatment of first-episode psychotic patients: a double-blind multicenter study. Risperidone Working Group. Schizophr. Bull..

[42] Sanger TM (1999). Olanzapine versus haloperidol treatment in first-episode psychosis. Am. J. Psychiatry.

[43] Lieberman JA (2003). Comparative efficacy and safety of atypical and conventional antipsychotic drugs in first-episode psychosis: a randomized, double-blind trial of olanzapine versus haloperidol. Am. J. Psychiatry.

[44] Dazzan P (2015). Magnetic resonance imaging and the prediction of outcome in first-episode schizophrenia: a review of current evidence and directions for future research. Schizophr. Bull..

[45] López-Gil X, Artigas F, Adell A (2009). Role of different monoamine receptors controlling MK-801-induced release of serotonin and glutamate in the medial prefrontal cortex: relevance for antipsychotic action. Int. J. Neuropsychopharmacol..

[46] Jauhar, S. et al. Determinants of treatment response in first-episode psychosis: an 18F-DOPA PET study. *Mol. Psychiatry*10.1038/s41380-018-0042-4 (2018). 10.1038/s41380-018-0042-4 PMC6331038 29679071

[47] Kay SR, Fiszbein A, Opler LA (1987). The positive and negative syndrome scale (PANSS) for schizophrenia. Schizophr. Bull..

[48] Gardner DM, Murphy AL, O’Donnell H, Centorrino F, Baldessarini RJ (2010). International consensus study of antipsychotic dosing. Am. J. Psychiatry.

[49] Andreasen NC (2005). Remission in schizophrenia: proposed criteria and rationale for consensus. Am. J. Psychiatry.

[50] Provencher SW (1993). Estimation of metabolite concentrations from localized in vivo proton NMR spectra. Magn. Reson. Med..

[51] Provencher, S. *LCModel & LCMgui User’s Manual*. http://s-provencher.com/lcm-manual.shtml (2015).

[52] Leucht S, Davis JM, Engel RR, Kane JM, Wagenpfeil S (2007). Defining ‘response’ in antipsychotic drug trials: recommendations for the use of scale-derived cutoffs. Neuropsychopharmacology.

