# Supplementary material for: Remission from antipsychotic treatment in first episode psychosis related to longitudinal changes in brain glutamate
Source: NPJ Schizophr. 2019 Aug 1;5:12. doi: 10.1038/s41537-019-0080-1 (PMC6672005; doi:10.1038/s41537-019-0080-1)
Supplement: Supplementary file 1 — Supplementary Information [file 41537_2019_80_MOESM1_ESM.docx]

# Supplementary Information


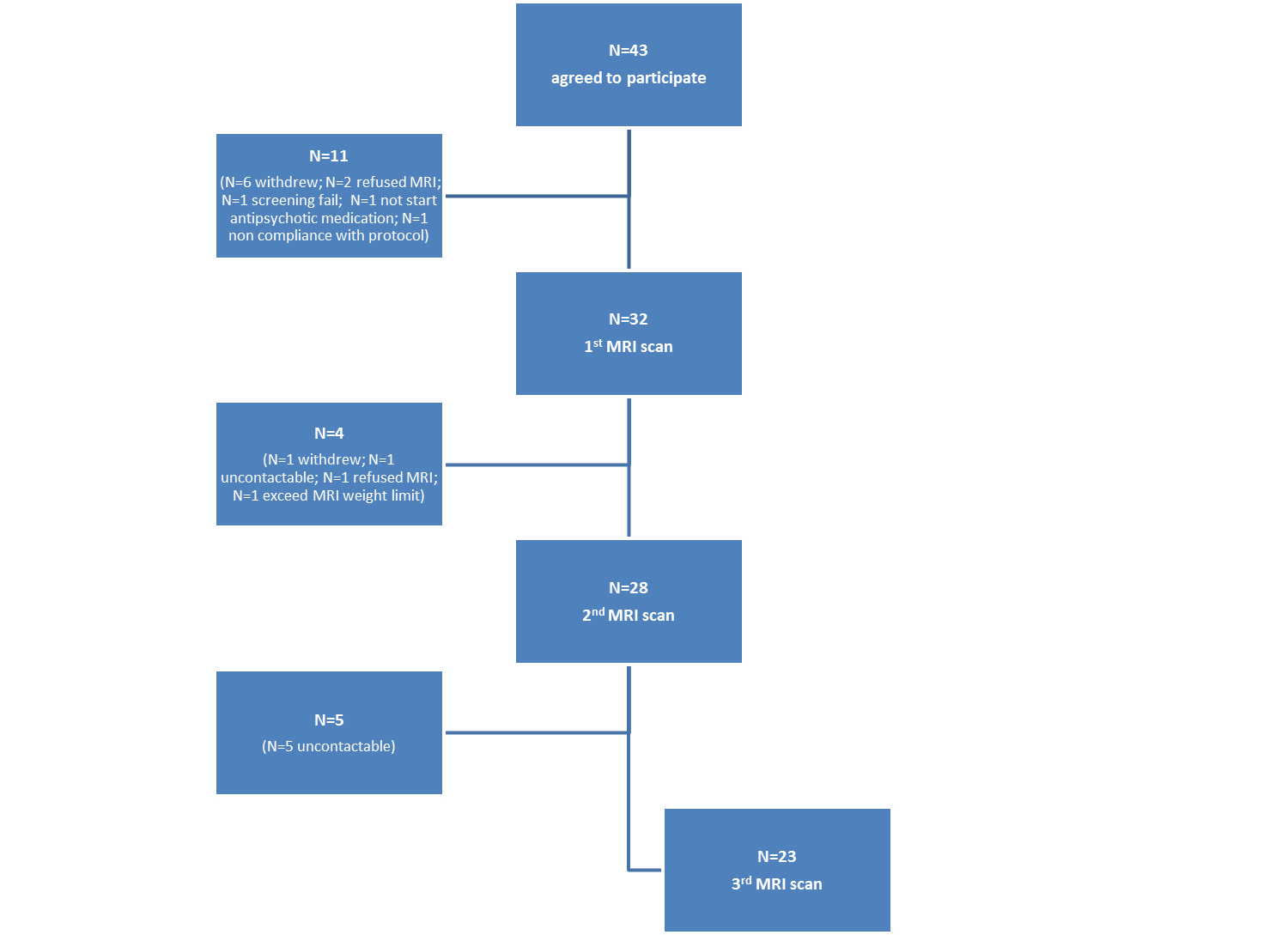


Figure 1 Flow diagram of the number of patients recruited into the study, and the number of drop outs at each scanning stage.

## Supplementary Notes

**Details on antipsychotic treatment**

Patients were clinically managed by local early intervention services specialised for patients with first episode psychosis. These teams employed an assertive approach, with relatively frequent assessments. Patients were encouraged to remain on antipsychotic medication, even when in remission. Most participants were initially treated with amisulpride, as they were taking part in a clinical trial involving this drug (OPTiMiSE). Once the clinical trial had been completed (typically after the 2^nd^ scan), the antipsychotic medication prescribed was selected by the patient’s clinical team.

At the time of the baseline scan, 4 patients were medication naïve and the mean duration of current antipsychotic treatment for the remaining patients was 12 days. All but one of the patients taking antipsychotic medication at baseline were receiving amisulpride. At the baseline scan patients received amisulpride at a dosage of 100-150mg (n=8), 200-250mg (n=9), 300mg (n=1) and risperidone at a dosage of 2mg (n=1).

At the 2^nd^ scan, patients had received antipsychotic treatment for a mean of 6 weeks, with most patients (n=16) receiving amisulpride (aripiprazole n=2, quetiapine n=2, olanzapine n=1, risperidone n=1). 19 patients reported full-adherence to medication in the period between baseline and the 2^nd^ scan. Of the remaining patients, 3 patients were delayed in initiating antipsychotic medication after the 1^st^ scan, but were adherent for at least 1 month preceding the 2^nd^ scan. One patient received antipsychotic medication for 1 month following the 1^st^ scan, but stopped 7 days before the 2^nd^ scan. At the 2^nd^ scan patients received amisulpride at a dosage of 100-150mg (n=2), 200-250mg (n=6), 300-400mg (n=8), aripiprazole at a dosage of 5mg (n=2), risperidone at a dosage of 4mg (n=1), olanzapine at a dosage of 5mg (n=1), and quetiapine at a dosage of 200mg (n=1) and 600mg (n=1).

At the 3^rd^ scan (9 month timepoint), the antipsychotic drug used to treat half of the patients had been changed from that at the 2^nd^ scan on one or more occasions, whereas 10 patients received amisulpride alone for the duration of the study. 15 patients were still taking antipsychotic medication at the 3^rd^ scan. The mean proportion of time that patients received medication between the 6 weeks and 9 month timepoints was 74%. At the 3^rd^ scan patients received amisulpride at a dosage of 200mg (n=1), 300-400mg (n=5), aripiprazole at a dosage of 5mg (n=2), risperidone at a dosage of 4mg (n=1), olanzapine at a dosage of 5mg (n=2), 7.5 (n=1), 10mg (n=1), 12mg (n=1), and quetiapine at a dosage of 200mg (n=1).

At both follow up points, none of the patients were taking more than one type of antipsychotic, and a minority of patients were taking anxiolytics (n=8), zopiclone (n=9), and antidepressants (n=1).

**Details on PANSS scores and demographic factors**

The Non-Remission group showed higher PANSS negative symptom scores at baseline compared to the Remission group (P=0.045), but did not differ on the other baseline clinical measures. At 6 weeks, the Non-Remission group had significantly higher PANSS positive (P=0.022), negative (P=0.008) and total scores (P=0.018), and significantly lower PSP scores (<P=0.045) compared to the Remission group. At 9 months, the Non-Remission group had significantly higher PANSS positive (<P=0.001), negative (P=0.028), general (P=0.003) and total scores (<P=0.001), and significantly lower PSP scores (P=0.048) than the Remission group (Table 1 in manuscript).

Healthy volunteers were more likely to be currently employed and had on average more years in education than the total patient sample, but these groups did not differ significantly on the other demographic variables (Table 1 in manuscript).

|  | Healthy Volunteers *n*=15 | FEP Patients  *n*=23 | Healthy Volunteers vs FEP Patients | Non Remission *n*=11 | Remission  *n*=12 | Non remission vs Remission |
| --- | --- | --- | --- | --- | --- | --- |
| Current smoker Y/N | 9/6 | 14/9 | X^2^ P=1.000 | 8/3 | 6/6 | X^2^ P=0.400 |
| Cigarettes/day mean (SD) | 4 (6) | 4 (5) | T (36)=-0.037; P=0.971 | 5 (5) | 4 (5) | T (21)=0.626; P=0.538 |
| Current alcohol drinker Y/N | 12/3 | 13/10 | X^2^ P=0.176 | 7/4 | 6/6 | X^2^ P=0.680 |
| Alcohol units/week mean (SD) | 5.9 (7.6) | 2.9 (4.6) | T (36)=1.527; P=0.135 | 2.6 (3.2) | 3.1 (5.7) | T (21)=-0.229; P=0.821 |
| Cannabis in last month Y/N | 7/8 | 8/15 | X^2^ P=0.514 | 5/6 | 3/9 | X^2^ P=0.400 |
| Cannabis Use  atleast 3x per month Y/N | 5/10 | 7/16 | X^2^ P=1.000 | 5/6 | 2/10 | X^2^ P=0.193 |
| Cocaine in last month Y/N | 3/12 | 4/19 | X^2^ P=1.000 | 2/9 | 2/10 | X^2^ P=1.000 |
| MDMA in last month Y/N | 2/13 | 1/22 | X^2^ P=0.550 | 0/11 | 1/11 | X^2^ P=1.000 |
| Ketamine in last month Y/N | 1/14 | 1/22 | X^2^ P=1.000 | 0/11 | 1/11 | X^2^ P=1.000 |

Table 1 Substance use at 9 months. No significant differences in substance use were found between the Healthy Volunteer and FEP (first episode psychosis) patient group, or between the Remission and Non-Remission groups.

|  | Healthy volunteers *n*=15 | Total patient group *n*=23 | Non-Remission  *n*=11 | Remission  *n*=12 |
| --- | --- | --- | --- | --- |
| ***Baseline 1H-MRS Scan*** | | | | |
| *Anterior cingulate cortex* | | | | |
| FWHM | 0.04 (0.01) | 0.04 (0.01) | 0.04 (0.01) | 0.04 (0.01) |
| SNR | 19.40 (4.26) | 20.04 (4.71) | 20.27 (5.53) | 19.83 (4.06) |
| Glx CRLB | 6.47 (0.51) | 7.17 (1.87) | 7.09 (2.02) | 7.25 (1.82) |
| Glu CRLB | 6.73 (1.16) | 6.83 (1.37) | 6.73 (1.49) | 6.92 (1.31) |
| NAA CRLB | 3.20 (0.41) | 3.48 (0.79) | 3.45 (0.93) | 3.50 (0.67) |
| Cho CRLB | 3.67 (0.62) | 3.30 (0.56) | 3.27 (0.47) | 3.33 (0.65) |
| mI CRLB | 5.40 (1.12) | 5.65 (1.40) | 5.36 (0.81) | 5.92 (1.78) |
| Cr CRLB | 3.00 (0.38) | 2.96 (0.56) | 2.91 (0.70) | 3.00 (0.43) |
| Included (n) | 15 | 23 | 11 | 12 |
| Failed QC (n) | 0 | 0 | 0 | 0 |
| *Left Thalamus* | | | | |
| FWHM | 0.05 (0.01) | 0.05 (0.01) | 0.05 (0.01) | 0.05 (0.01) |
| SNR | 18.33 (4.32) | 18.61 (1.85) | 18.91 (1.38) | 18.33 (2.23) |
| Glx CRLB | 8.93 (3.13) | 10.22 (2.35) | 10.91 (1.97) | 9.58 (2.57) |
| Glu CRLB | 8.73 (1.67) | 9.09 (1.83) | 9.18 (0.87) | 9.00 (2.45) |
| NAA CRLB | 3.33 (0.98) | 3.30 (0.56) | 3.36 (0.50) | 3.25 (0.62) |
| Cho CRLB | 3.33 (0.62) | 3.57 (0.59) | 3.45 (0.52) | 3.67 (0.65) |
| mI CRLB | 7.13 (2.26) | 7.57 (1.47) | 7.55 (1.21) | 7.58 (1.73) |
| Cr CRLB | 3.13 (0.35) | 3.13 (.34) | 3.18 (0.40) | 3.08 (0.29) |
| Included (n) | 15 | 23 | 11 | 12 |
| Failed QC (n) | 0 | 0 | 0 | 0 |
| ***6 weeks 1H-MRS Scan*** | | | | |
| *Anterior cingulate cortex* | | | | |
| FWHM | 0.04 (0.01) | 0.04 (0.01) | 0.04 (0.01) | 0.04 (0.01) |
| SNR | 17.93 (3.95) | 19.57 (3.93) | 19.36 (4.30) | 19.75 (3.74) |
| Glx CRLB | 7.00 (1.00) | 6.78 (1.28) | 6.91 (1.38) | 6.67 (1.23) |
| Glu CRLB | 7.53 (1.06) | 7.09 (1.47) | 7.36 (1.69) | 6.83 (1.27) |
| NAA CRLB | 3.40 (0.63) | 3.27 (0.46) | 3.18 (0.40) | 3.33 (0.65) |
| Cho CRLB | 3.67 (0.90) | 3.22 (0.60) | 3.18 (0.40) | 3.25 (0.75) |
| mI CRLB | 5.53 (0.64) | 5.26 (0.86) | 5.27 (0.79) | 5.25 (0.97) |
| Cr CRLB | 3.13 (0.35) | 3.00 (0.52) | 3.00 (0.63) | 3.00 (0.43) |
| Included (n) | 15 | 23 | 11 | 12 |
| Failed QC (n) | 0 | 0 | 0 | 0 |
| *Left Thalamus* | | | | |
| FWHM | 0.05 (0.01) | 0.05 (0.01) | 0.05 (0.01) | 0.05 (0.01) |
| SNR | 18.53 (3.25) | 17.78 (2.33) | 17.91 (2.51) | 17.67 (2.27) |
| Glx CRLB | 10.67 (3.35) | 10.91 (2.66) | 11.36 (2.54) | 10.50 (2.81) |
| Glu CRLB | 9.87 (3.60) | 10.09 (1.50) | 10.00 (1.41) | 10.17 (1.64) |
| NAA CRLB | 3.40 (0.63) | 3.39 (0.58) | 3.18 (0.40) | 3.58 (0.67) |
| Cho CRLB | 3.40 (0.63) | 3.65 (0.49) | 3.64 (0.50) | 3.67 (0.49) |
| mI CRLB | 7.07 (1.16) | 7.61 (1.53) | 7.64 (1.86) | 7.58 (1.24) |
| Cr CRLB | 3.27 (0.46) | 3.35 (0.49) | 3.36 (0.50) | 3.33 (0.49) |
| Included (n) | 15 | 23 | 11 | 12 |
| Failed QC (n) | 0 | 0 | 0 | 0 |
| ***9 month 1H-MRS Scan*** | | | | |
| *Anterior cingulate cortex* | | | | |
| FWHM | 0.04 (0.01) | 0.04 (0.01) | 0.04 (0.01) | 0.04 (0.01) |
| SNR | 18.93 (3.33) | 19.09 (4.12) | 18.64 (4.18) | 19.50 (4.21) |
| Glx CRLB | 6.93 (1.10) | 7.26 (1.48) | 7.18 (1.83) | 7.33 (1.15) |
| Glu CRLB | 7.13 (1.13) | 7.30 (1.58) | 7.36 (1.50) | 7.25 (1.71) |
| NAA CRLB | 3.27 (0.46) | 3.65 (0.71) | 3.55 (0.69) | 3.75 (0.75) |
| Cho CRLB | 3.60 (0.51) | 3.52 (0.59) | 3.55 (0.52) | 3.50 (0.67) |
| mI CRLB | 5.80 (0.86) | 5.52 (1.16) | 5.55 (1.37) | 5.50 (1.00) |
| Cr CRLB | 3.07 (0.26) | 3.09 (0.42) | 3.09 (0.54) | 3.08 (0.29) |
| Included (n) | 15 | 23 | 11 | 12 |
| Failed QC (n) | 0 | 0 | 0 | 0 |
| *Left Thalamus* | | | | |
| FWHM | 0.05 (0.01) | 0.06 (0.01) | 0.05 (0.01) | 0.06 (0.01) |
| SNR | 18.53 (3.93) | 16.52 (3.63) | 16.64 (4.54) | 16.42 (2.75) |
| Glx CRLB | 11.07 (3.92) | 12.17 (6.59) | 10.55 (2.16) | 13.67 (8.80) |
| Glu CRLB | 9.73 (3.13) | 10.0 (3.83) | 9.36 (1.91) | 10.58 (5.02) |
| NAA CRLB | 3.27 (0.59) | 3.26 (0.54) | 3.27 (0.65) | 3.25 (0.45) |
| Cho CRLB | 3.87 (1.55) | 3.87 (0.81) | 3.91 (0.83) | 3.83 (0.83) |
| mI CRLB | 7.80 (2.01) | 7.59 (1.53) | 7.50 (1.58) | 7.67 (1.56) |
| Cr CRLB | 3.27 (0.80) | 3.61 (0.72) | 3.55 (0.69) | 3.67 (0.78) |
| Included (n) | 14 | 14 (Glu, Glx, mI) | 10 (mI) | 11 (Glu, Glx) |
| Failed QC (n) | 1 (Glx) | 1 (Glu, Glx, mI) | 1 (mI) | 1 (Glu, Glx) |

Table 2 1H-MRS quality measures. Data are presented as mean (SD). There were no significant differences (P < 0.05) between Healthy Volunteers and Patients, or Remission and Non-Remission groups. FWHM: Spectral line-width as full-width at half-maximum in parts per million; SNR: signal to noise ratio; CRLB Cramer Rao Lower Bounds.

|  | Repeated measures ANOVA | Post hoc (time): Repeated measures ANOVA in one group only | Post hoc (group): One way ANOVA between groups at 9 month timepoint |
| --- | --- | --- | --- |
| *ACC Glx* |  |  |  |
| Remission *n*=12 | Group: F(1,21)=0.010, P=0.921  Time: F(2,42)=0.095, P=0.910  Interaction: F(2,42)=0.332, P=0.719 | - | - |
| Non-Remission *n*=11 |  | - |  |
|  |  |  |  |
| Healthy Volunteers *n*=15 | Group: F(1,36)=0.078, P=0.781  Time: F(2,72)=0.210, P=0.811  Interaction: F(2,72)=0.705, P=0.498 | - | - |
| Total Patient group *n*=23 |  | - |  |
|  |  |  |  |
| *ACC Glutamate* |  |  |  |
| Remission *n*=12 | Group: F(1,21)=0.209, P=0.652  Time: F(1.5,31.5)=0.456, P=0.582  Interaction: F(1.5,31.5)=0.072, P=0.882 | - | - |
| Non-Remission *n*=11 |  | - |  |
|  |  |  |  |
| Healthy Volunteers *n*=15 | Group: F(1,36)=0.146, P=0.705  Time: F(1.7,60.1)=0.530, P=0.559  Interaction: F(1.7,60.1)=1.672, P=0.200 | - | - |
| Total Patient group *n*=23 |  | - |  |
|  |  |  |  |
| *Left thalamus Glx* |  |  |  |
| Remission *n*=11 | Group: F(1,20)=0.121, P=0.731  Time: F(2,40)=2.541, P=0.091  **Interaction: F(2,40)=4.337, P=0.020*** | F(2,20)=1.849, P=0.183 | **F(1,20)=5.244; P=0.033*** |
| Non-Remission *n*=11 |  | **F(2,20)=6.183, P=0.008**** |  |
|  |  |  |  |
| Healthy Volunteers *n*=14 | Group: F(1,34)=0.021, P=0.887  Time: F(2,68)=2.429, P=0.096  Interaction: F(2,68)=0.312, P=0.733 | - | - |
| Total Patient group *n*=22 |  | *-* |  |
|  |  |  |  |
| *Left thalamus Glutamate* | |  |  |
| Remission *n*=11 | Group: F(1,20)=0.036, P=0.852  **Time: F(2,40)=7.306, P=0.002****  Interaction: F(2,40)=1.310, P=0.281 | F(2,20)=2.754, P=0.088 | - |
| Non-Remission *n*=11 |  | **F(1.3,13.3)=7.961, P=0.010**** |  |
|  |  |  |  |
| Healthy Volunteers *n*=15 | Group: F(1,35)=0.136, P=0.714  **Time: F(2,70)=3.753, P=0.028***  Interaction: F(2,70)=1.364, P=0.262 | F(2,28)=0.606, P=0.553 | - |
| Total Patient group *n*=22 |  | **F(2,42)=7.200, P=0.002**** |  |
|  |  |  |  |

Table 3 Repeated measures ANOVA statistics assessing glutamatergic metabolites over time in relation to remission status or clinical group. Analyses conducted for Glx and Glutamate in the anterior cingulate cortex (ACC) and left thalamus, in the i) Remission and Non-Remission groups, and ii) Healthy Volunteer and total Patient groups. Significant repeated measures ANOVA analyses are explored further by post-hoc tests. One way ANOVA analyses between groups is shown for the 9 month timepoint only, as significant results are not found at other timepoints. Significant results are highlighted in bold and represented by *=P<0.05, **= P<0.01.


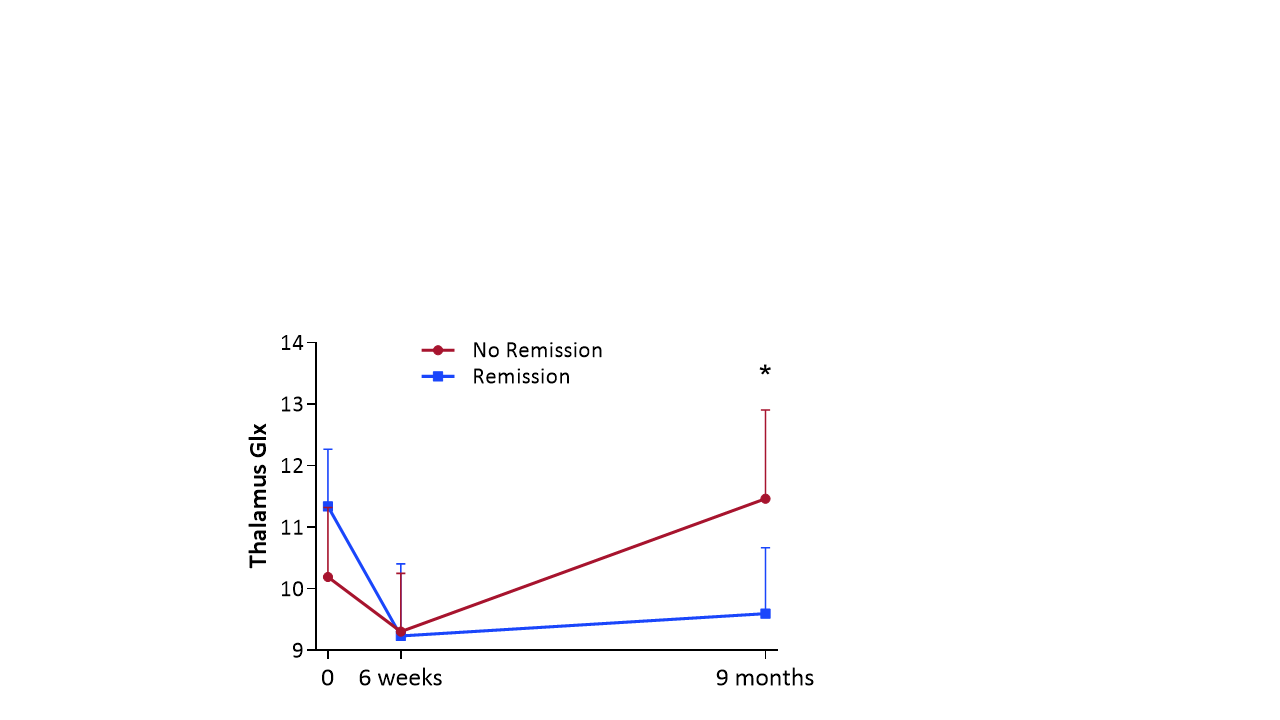


Figure 2 Mean CSF-corrected Glx in the thalamus at Baseline, 6 weeks and 9 months, in the Remission and Non-Remission groups, excluding patients who were medication adherent less than 75% of the time. *Represents higher thalamic Glx levels in the Non-Remission group compared to the Remission group at 9 months (P=0.043). Error bars represent within-subjects standard deviation.

For Glx in the thalamus (Supplementary Figure 2 above), when analysis was restricted to the adherent subgroup (excluding patients who reported being adherent to antipsychotic medication less than 75% of the time), the interaction between remission status and time remained significant (F(2,26)=4.698, *P*=0.018, repeated measures ANOVA) and there was also a significant main effect of time (F(2,26)=5.300, *P*=0.012) but not remission status (F(1,13)=0.130, *P*=0.724). As in the larger sample, Glx levels were significantly higher in the Non-Remission than the Remission group at 9 months (F(1,13)=5.053, P=0.043, one way ANOVA), related to a main effect of time in the Non-Remission group (F(2,12)=3.894, P=0.050, repeated measures ANOVA). Time was also significant in the Remission group (F(2,14)=6.435, P=0.010, repeated measures ANOVA) due to a decrease in Glx between baseline and 6 weeks (*P*=0.037, Bonferroni-corrected pairwise comparisons).

|  | **PANSS positive score** | **PANSS negative score** | **PANSS general score** | **PANSS total score** | **PSP** |
| --- | --- | --- | --- | --- | --- |
| **Thalamus Glutamate n=22** | *r*=.210, *P*=0.348 | *r*=.005, *P*=0.983 | *r*=.297, *P*=0.179 | *r*=.218, *P*=0.330 | *r*=.127, *P*=0.573 |
| **ACC Glx n=23** | *r*=-.100, *P*=0.651 | *r*=.035, *P*=0.875 | *r*=.201, *P*=0.358 | *r*=.049, *P*=0.823 | *r*=.234, *P*=0.282 |
| **ACC Glutamate n=23** | *r*=-.079, *P*=0.720 | *r*=.142, *P*=0.518 | *r*=.045, *P*=0.837 | *r*=-.037, *P*=0.866 | *r*=.297, *P*=0.169 |

Table 4 Relationship between percentage change in metabolite level, and percentage change in clinical scores between the baseline and the 9 month timepoint in patients (Pearson’s bivariate correlation).

| Healthy Volunteers vs Total Patient group | | |
| --- | --- | --- |
| ACC | Metabolites x Group | F(3,108)=0.542, P=0.655 |
|  | Time x Group | F(2,72)=0.447, P=0.641 |
|  | Metabolites x Time | F(4.4,158.3)=0.552, P=0.714 |
|  | Metabolites x Time x Group | F(4.4,158.3)=0.634, P=0.654 |
| Thalamus | Metabolites x Group | F(2.3,80)=0.602, P=0.572 |
|  | Time x Group | F(2,70)=3.520, P=0.010** |
|  | Metabolites x Time | F(4.2,146.4)=0.333, P=0.863 |
|  | Metabolites x Time x Group | F(4.2,146.4)=1.313, P=0.267 |
| Remission vs Non-Remission | | |
| ACC | Metabolites x Group | F(3,63)=0.340, P=0.796 |
|  | Time x Group | F(2,42)=0.133, P=0.876 |
|  | Metabolites x Time | F(6,126)=0.439, P=0.851 |
|  | Metabolites x Time x Group | F(6,126)=0.214, P=0.972 |
| Thalamus | Metabolites x Group | F(3,60)=0.028, P=0.994 |
|  | Time x Group | F(2,40)=0.452, P=0.639 |
|  | Metabolites x Time | F(3.8,75.3)=0.792, P=0.527 |
|  | Metabolites x Time x Group | F(3.8,75.3)=0.265, P=0.890 |

Table 5 Repeated measures MANOVA analyses were conducted on other 1H-MRS metabolite concentrations (N-acetyl-aspartate, creatine, myo-inositol, choline) between i) Healthy Volunteers vs Patients and ii) Remission vs Non-Remission across 3 timepoints. **=P<0.01. Post-hoc tests did not find significant effects when diagnostic groups and timepoints were analysed separately.
